# Contrasting MRI patterns in early infant human parechovirus CNS infection: a brief report

**DOI:** 10.1007/s00431-026-06955-x

**Published:** 2026-05-05

**Authors:** Aldo Naselli, Francesca Tota, Stefania Ielo, Valentina Folgheraiter, Umberto Rozzanigo, Giovanni Lorenzin, Giuliana Marchio, Anna Rosati

**Affiliations:** 1 Neonatal Intensive Care Unit, S. Chiara Hospital, Largo Medaglie d’oro, Trento, 38122 Italy; 2 Paediatric Unit, S. Chiara Hospital, Largo Medaglie d’oro, Trento, 38122 Italy; 3 Neuroradiology and Interventional Radiology Unit, S. Chiara Hospital, Largo Medaglie d’oro, Trento, 38122 Italy; 4 Microbiology Unit, S. Chiara Hospital, Largo Medaglie d’oro, Trento, 38122 Italy; 5 Pediatric Neuropsychiatry Unit, S. Chiara Hospital, Largo Medaglie d’oro, Trento, 38122 Italy

**Keywords:** Human parechovirus, Neonatal encephalitis, MRI, White matter injury, Meningitis

## Abstract

Human parechovirus (HPeV), particularly genotype A3, is a recognized cause of sepsis-like illness and central nervous system (CNS) infection in neonates and young infants. Brain magnetic resonance imaging (MRI) plays a key role in identifying CNS involvement, although neuroradiological findings may be heterogeneous. We report two infants with PCR-confirmed HPeV CNS infection presenting at 10 and 40 days of life, respectively, with contrasting MRI patterns. The first neonate showed the typical phenotype of bilateral fronto-parietal and periventricular white matter diffusion restriction despite minimal cerebrospinal fluid (CSF) pleocytosis. The second infant demonstrated isolated diffuse leptomeningeal enhancement without parenchymal lesions. Both patients experienced rapid clinical recovery and remained seizure-free; neurodevelopment was normal at follow-up in the case with white matter involvement. These observations highlight the spectrum of MRI findings in early infant HPeV CNS infection and reinforce that normal or near-normal CSF parameters do not exclude significant CNS involvement. Early MRI, combined with molecular testing, may improve diagnostic accuracy and inform follow-up strategies.

*Conclusion*: These observations highlight the spectrum of MRI findings in early infant HPeV CNS infection and reinforce that normal or near-normal CSF parameters do not exclude significant CNS involvement. Early MRI, combined with molecular testing, may improve diagnostic accuracy and inform follow up strategies.

**Table:** 

** What is Known: **
• *Human parechovirus (HPeV) CNS infection in young infants is commonly associated with white-matter diffusion abnormalities on MRI and minimal or absent CSF pleocytosis*.
• *MRI is a key diagnostic tool in identifying CNS involvement, although neuroradiological findings may be heterogeneous*.
** What is New: **
• *This report describes two infants with HPeV CNS infection showing contrasting MRI patterns, including isolated leptomeningeal enhancement without parenchymal lesions*.
• *These findings broaden the recognized neuroradiological spectrum of HPeV infection and highlight the diagnostic value of MRI even in clinically mild cases*.

**Supplementary information:**

The online version contains supplementary material available at 10.1007/s00431-026-06955-x.

## Background

Human parechovirus (HPeV), particularly genotype A3, is an established cause of sepsis-like illness and central nervous system (CNS) infection in neonates and young infants, especially during seasonal outbreaks in late summer and autumn [1–4]. A distinctive diagnostic feature is the frequent absence of cerebrospinal fluid (CSF) pleocytosis despite confirmed CNS involvement, which may delay recognition if molecular testing is not routinely performed [3–5].

Brain magnetic resonance imaging (MRI) has emerged as a key tool in identifying CNS involvement. The most characteristic imaging pattern described in HPeV encephalitis consists of bilateral fronto-parietal and periventricular white matter diffusion restriction with radial distribution

toward the cortex, a phenotype mainly reported in neonates and associated in some cohorts with adverse neurodevelopmental outcomes [6–8]. However, MRI findings may be heterogeneous, and atypical or milder patterns are increasingly recognized.

We describe two infants with PCR-confirmed HPeV CNS infection presenting at different ages within early infancy and showing contrasting neuroradiological phenotypes, aiming to highlight the diagnostic and follow-up implications of this variability.

## Case presentations

### Case 1

A male neonate born at 40+1 weeks of gestation via cesarean section for placental abruption had Apgar scores of 9 and 10 at 1 and 5 minutes, respectively, did not require resuscitation at birth, and showed no clinical signs of neonatal encephalopathy. The early neonatal course was uneventful. Maternal serology was unremarkable except for incomplete intrapartum antibiotic prophylaxis for Group B Streptococcus. He was discharged at 3 days of life in good condition. At 10 days of life, he developed poor feeding, irritability, and fever (38.6 °C), prompting admission to the Pediatric Department of Trento Hospital.

**Fig. 1:**
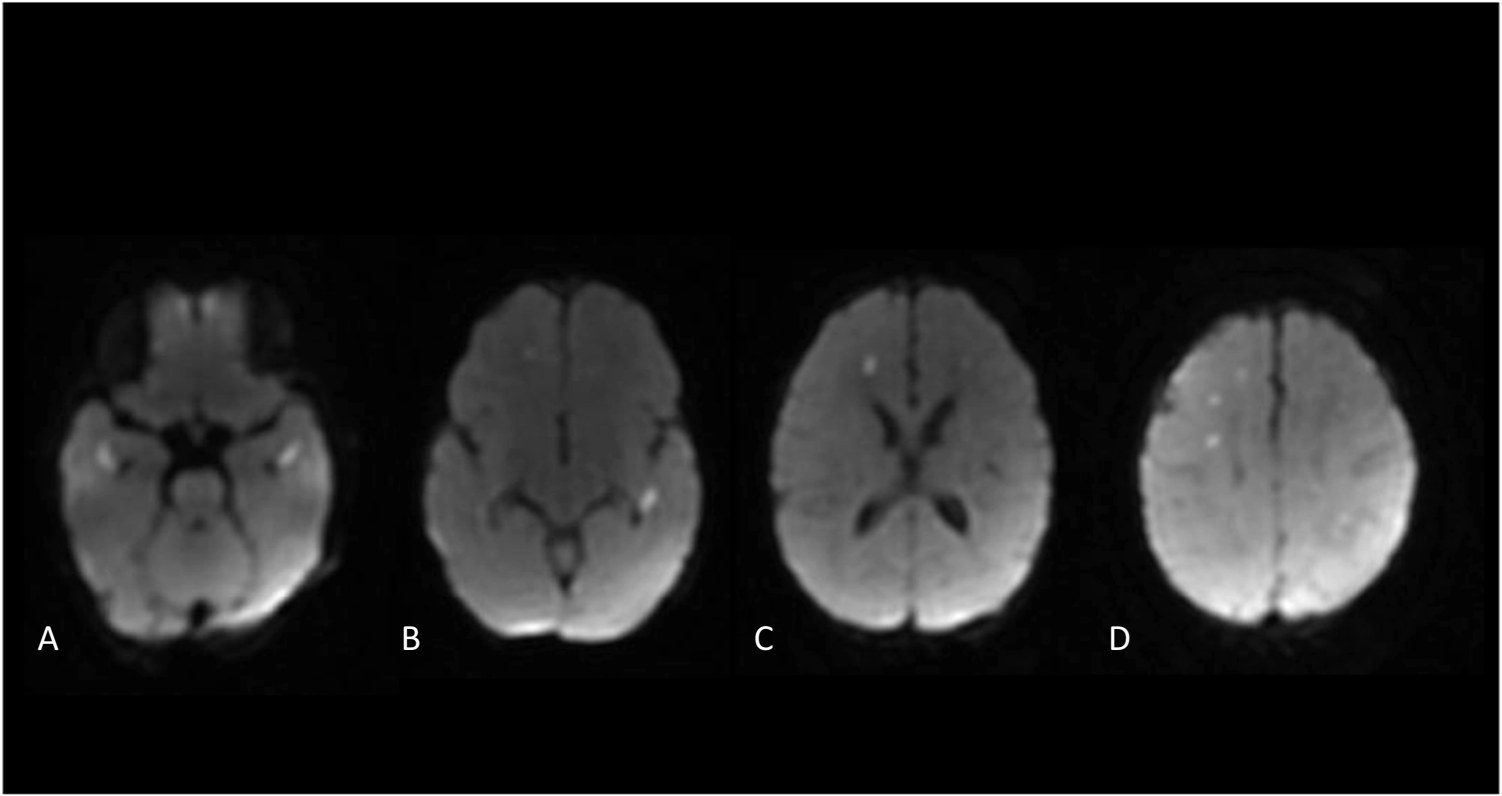
DWI images at different brain levels (pons, mesencephalon, basal ganglia, and corona radiata) of the same patient demonstrate multiple foci of restricted diffusion, confirmed on ADC maps, involving the periventricular and subcortical white matter and distributed along the course of deep medullary veins

On admission, he appeared lethargic but responsive, with normal tone and reflexes. Initial laboratory tests revealed a normal leukocyte count (6.5×10⁹/L) and C-reactive protein (<2.9 mg/L). Lumbar puncture showed clear CSF with 2 cells/µL, protein 75 mg/dL, and glucose 54 mg/dL. Culture was sterile. PCR testing of cerebrospinal fluid was negative for common bacterial and viral pathogens (HSV, VZV, CMV, and enterovirus) but positive for human parechovirus RNA. Viral genotyping was not performed; therefore, the specific HPeV genotype could not be determined. Brain MRI was performed on a 1.5-T scanner and included axial and sagittal T1-weighted TSE, axial T2-weighted TSE and T2*-GRE, FLAIR, diffusion-weighted imaging (DWI) with apparent diffusion coefficient (ADC) maps, and post-contrast T1-weighted TSE sequences after intravenous gadolinium administration. The DWI sequence demonstrated multiple small punctate lesions (Fig. 1A–D) in the supratentorial deep white matter bilaterally, sparing the basal ganglia, brainstem, and cerebellum. All the lesions were characterized by diffusion restriction in the ADC maps, spontaneous hyperintensity in the T1-weighed images, without significant contrast enhancement. All MRI studies were independently reviewed by an experienced pediatric neuroradiologist.

He was treated empirically with intravenous amoxicillin and netilmicin until bacterial cultures were reported and confirmed negative. Supportive management included parenteral fluids and careful neurological monitoring. He remained seizure-free throughout hospitalization. Gradual improvement in feeding and reactivity was observed after day 4 of hospitalization. He was discharged on day 12 of life in good clinical condition. Follow-up at one and two months revealed normal neurodevelopmental milestones, adequate weight gain, and normal tone. At 2, 6, 9, and 24 months, neuropsychiatric evaluation confirmed age-appropriate behavior, visual tracking, and normal reflexes. MRI findings were consistent with mild white matter injury. At follow-up, no neurodevelopmental impairment was detected.

### Case 2

A 40-day-old male born at term (39 weeks) via vaginal delivery, presented to the emergency department of Cavalese Hospital with fever, irritability, and feeding difficulties. Physical examination revealed a pale, mottled infant with tachycardia (200 bpm) and prolonged capillary refill. He was transferred to the neonatal intensive care unit (NICU) of Trento for further evaluation.

Initial investigations showed normal CRP (3.7 mg/L) and mild leukopenia (2.9×10⁹/L) with neutropenia (0.4 × 10⁹/L). CSF analysis revealed normal glucose and protein, and bacterial cultures were negative. HPeV RNA was detected in cerebrospinal fluid by polymerase chain reaction (PCR). Viral genotyping was not performed; therefore, the specific HPeV genotype could not be determined. EEG demonstrated mild asymmetry with increased delta activity over the left centro-parietal regions but no epileptiform discharges. Cranial ultrasound was normal. Brain MRI was performed on a 1.5-T scanner and included axial and sagittal T1-weighted TSE, axial T2-weighted TSE, SWI, FLAIR, diffusion-weighted imaging (DWI) with apparent diffusion coefficient (ADC) maps, and post-contrast 3D T1-weighted sequences after intravenous gadolinium administration.

**Fig. 2:**
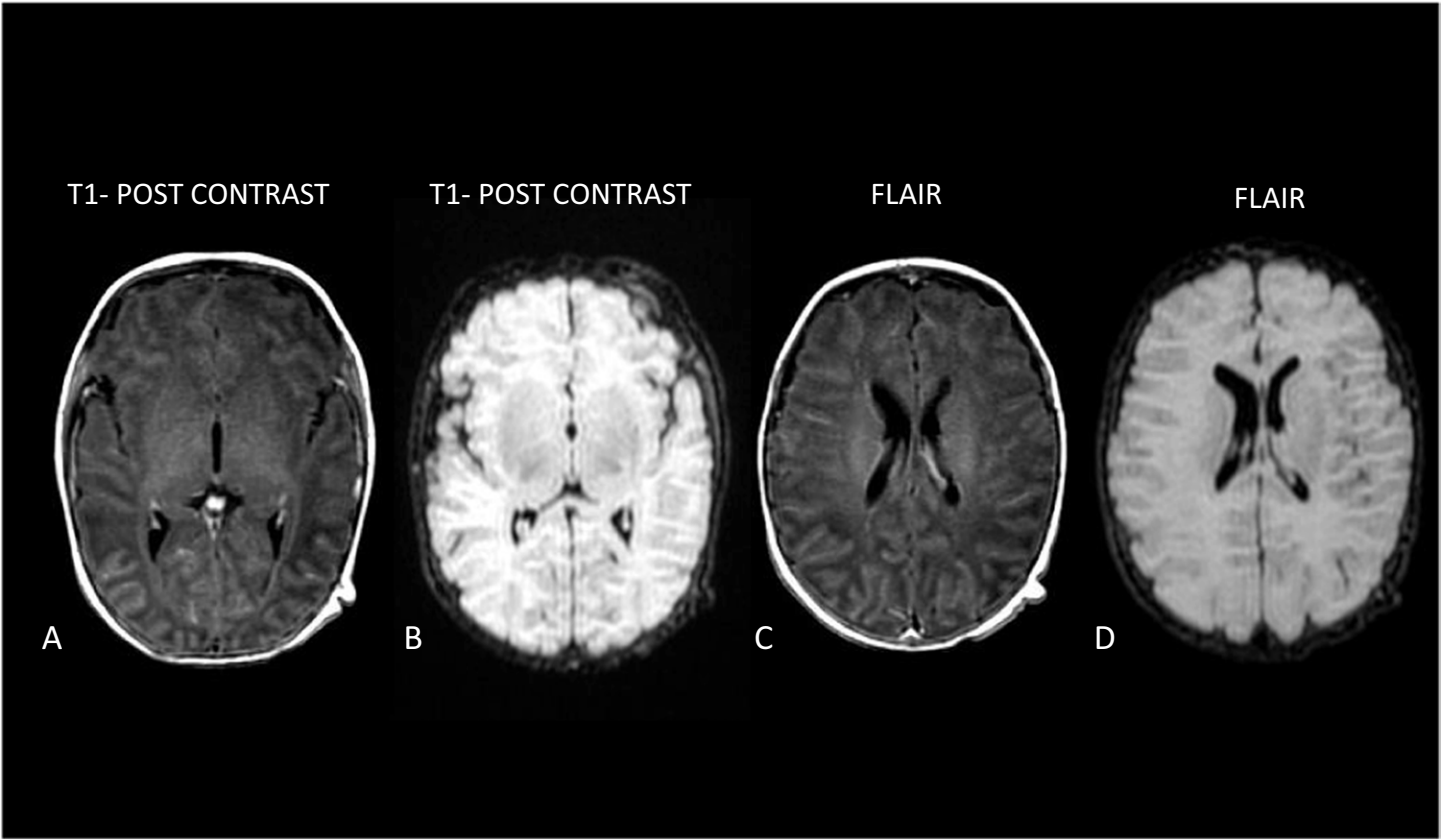
**A**, **B** Axial post-contrast T1-weighted images demonstrating diffuse leptomeningeal enhancement. **C**, **D** Corresponding axial FLAIR images obtained before contrast administration showing absence of sulcal hyperintensity or parenchymal abnormalities. Axial multiplanar reconstructions were derived from a sagittal 3D fat-suppressed FLAIR sequence obtained before contrast administration. Diffusion-weighted imaging showed no areas of restricted diffusion. ADC maps are not shown

Post-contrast T1-weighted images (Fig. 2A and B) showed diffuse leptomeningeal enhancement without parenchymal lesions. The corresponding FLAIR images obtained before contrast administration (Fig. 2C and D) showed no sulcal hyperintensity. Diffusion-weighted imaging showed no areas of restricted diffusion. All MRI studies were independently reviewed by an experienced pediatric neuroradiologist. He received empirical ampicillin and netilmicin, which were discontinued once bacterial cultures were negative. Supportive therapy, including oxygen and fluids, was continued. No seizures were observed. Clinical improvement was noted within three days, with normalization of tone and feeding. Echocardiography revealed a structurally normal heart. The patient was discharged in good general condition on day 4 of hospitalization. Follow-up MRI after 10 days showed resolution of meningeal enhancement. In our patient, this imaging pattern was associated with a mild clinical course, rapid recovery, and complete radiological resolution; however, given the single-case observation and the limited follow-up duration, no firm prognostic conclusions can be drawn.

## Discussion

In recent years, MRI has become central in the diagnostic evaluation of suspected HPeV CNS infection in early infancy. Cohort studies and neuroradiological series have consistently described a characteristic phenotype of bilateral deep white matter diffusion restriction, particularly in association with HPeV-A3 infection [7, 8]. This pattern is considered highly suggestive in neonates presenting with sepsis-like illness and minimal CSF abnormalities.

Our first case, occurring at 10 days of life, reflects this well-recognized imaging presentation. Despite minimal CSF pleocytosis, bilateral periventricular and fronto-parietal diffusion restriction was evident, underscoring the importance of combining molecular diagnostics with early neuroimaging. The absence of CSF pleocytosis has been repeatedly emphasized as a distinctive feature of HPeV CNS infection in infants younger than three months [3–5], supporting routine PCR testing in this age group.

Longitudinal data suggest that infants with parenchymal white matter involvement may be at increased risk of later neurodevelopmental impairment compared with those without significant MRI abnormalities [6, 7]. Although placental abruption occurred at delivery, the reassuring perinatal parameters and absence of neonatal encephalopathy make hypoxic–ischemic injury unlikely as an alternative explanation for the MRI findings. Even though our patient showed normal development up to 24 months, structured follow-up remains advisable in cases with documented white matter injury.

In contrast, the second case, presenting at 40 days of life, demonstrated isolated leptomeningeal enhancement without parenchymal diffusion abnormalities. This pattern is less frequently emphasized in the literature, where most MRI-focused reports highlight white matter injury rather than isolated meningeal involvement [7–9]. In our patient, meningeal enhancement was associated with a mild clinical course and rapid radiological resolution. While firm prognostic conclusions cannot be drawn from a single case, this observation supports the concept that HPeV CNS infection may encompass a broader neuroradiological spectrum than traditionally described.

Age at presentation may contribute to this variability. European epidemiological data indicate that the most severe forms predominantly occur in the neonatal period, whereas infections later in early infancy may follow a milder course [3, 4, 6]. Our two cases, occurring at 10 and 40 days of life, respectively, align with this observation, although larger studies are required to clarify potential age-dependent imaging patterns.

Rather than proposing a novel imaging entity, this brief report illustrates two contrasting manifestations within the recognized spectrum of HPeV CNS infection [10, 11]. Awareness of both classic white matter injury and isolated leptomeningeal enhancement may facilitate timely diagnosis, appropriate counseling, and tailored neurodevelopmental follow-up in young infants with sepsis-like illness and negative routine CSF findings.

This case report was prepared in accordance with the CARE guidelines for case reports.

## Conclusions

Human parechovirus infection represents an important and often underrecognized cause of central nervous system involvement in neonates and young infants. Our two cases illustrate the marked heterogeneity of neuroradiological presentations, ranging from classic white matter diffusion abnormalities to isolated meningeal enhancement without parenchymal lesions. These observations underscore that normal or near-normal cerebrospinal fluid findings do not exclude CNS infection and highlight the critical role of PCR testing in establishing the diagnosis. Early brain MRI may provide valuable diagnostic and prognostic information, supporting risk stratification even in clinically mild cases. Although both infants in our report showed favorable early clinical outcomes, longer term neurodevelopmental follow-up remains essential, particularly in those with white matter involvement. Awareness of both typical and atypical MRI patterns may support earlier diagnosis and structured neurodevelopmental follow-up in young infants with suspected HPeV CNS infection.

## Supplementary information

Below is the link to the electronic supplementary material.

ESM 1 (DOCX 12.8 KB)

## Data Availability

No datasets were generated or analysed during the current study.
