# Supplementary material for: Contrasting MRI patterns in early infant human parechovirus CNS infection: a brief report
Source: Eur J Pediatr. 2026 May 5;185(6):347. doi: 10.1007/s00431-026-06955-x (PMC13139296; doi:10.1007/s00431-026-06955-x)
Supplement: Supplementary file 1 — (DOCX 12.8 KB) [file 431_2026_6955_MOESM1_ESM.docx]

**Table 1.** Ultra-compact comparison of two infants with human parechovirus (HPeV) CNS infection: clinical presentation, CSF findings, and neuroradiological phenotype.

| **Variable** | **Case 1 (Neonate)** | **Case 2 (Young Infant)** |
| --- | --- | --- |
| Age at onset | 10 days | 40 days |
| Presentation | Fever, irritability, poor feeding | Sepsis-like illness with fever, irritability, poor feeding |
| CSF profile | Minimal pleocytosis (2 cells/µL); mildly increased protein | Normal CSF (no pleocytosis reported) |
| Diagnosis | CSF PCR positive for HPeV RNA | CSF PCR positive for HPeV RNA |
| Key MRI finding | Bilateral periventricular/fronto-parietal white-matter diffusion restriction | Isolated diffuse leptomeningeal enhancement; no parenchymal lesions |
| Early outcome | Rapid recovery; no seizures; normal neurodevelopment at follow-up (up to 24 months) | Rapid recovery; no seizures; MRI normalization at 10 days |

**Teaching point:** HPeV CNS infection may present with opposite neuroradiological phenotypes (white-matter diffusion restriction vs isolated leptomeningeal enhancement) despite absent or minimal CSF pleocytosis.

**Abbreviations:** CNS, central nervous system; CSF, cerebrospinal fluid; HPeV, human parechovirus; MRI, magnetic resonance imaging; PCR, polymerase chain reaction.
